# Auranofin Is Highly Efficacious against *Toxoplasma gondii In Vitro* and in an *In Vivo* Experimental Model of Acute Toxoplasmosis

**DOI:** 10.1371/journal.pntd.0002973

**Published:** 2014-07-31

**Authors:** Rosa M. Andrade, Juan D. Chaparro, Edmund Capparelli, Sharon L. Reed

**Affiliations:** 1 Department of Medicine, University of California, San Diego, San Diego, California, United States of America; 2 Department of Pediatrics, University of California, San Diego, San Diego, California, United States of America; 3 Department of Pathology, University of California, San Diego, San Diego, California, United States of America; Pollastri, Northeastern University, United States of America

## Abstract

**Background:**

The mainstay of toxoplasmosis treatment targets the folate biosynthetic pathways and has not changed for the last 50 years. The activity of these chemotherapeutic agents is restricted to one lifecycle stage of *Toxoplasma gondii*, they have significant toxicity, and the impending threat of emerging resistance to these agents makes the discovery of new therapies a priority. We now demonstrate that auranofin, an orally administered gold containing compound that was FDA approved for treatment of rheumatoid arthritis, has activity against *Toxoplasma gondii in vitro* (IC_50_ = 0.28 µM) and *in vivo* (1 mg/kg).

**Methods/Principal Findings:**

Replication within human foreskin fibroblasts of RH tachyzoites was inhibited by auranofin. At 0.4 µM, auranofin inhibited replication, as measured by percent infected fibroblasts at 24 hrs, (10.94% *vs.* 24.66% of controls; *p* = 0.0003) with no effect on parasite invasion (16.95% *vs.* 12.91% *p* = 0.4331). After 18 hrs, 62% of extracellular parasites treated with auranofin were non-viable compared to control using an ATP viability assay (*p* = 0.0003). *In vivo*, a previously standardized chicken embryo model of acute toxoplasmosis was used. Fourteen day old chicken embryos were injected through the chorioallantoic vein with 1×10^4^ tachyzoites of the virulent RH strain. The treatment group received one dose of auranofin at the time of inoculation (1 mg/kg estimated body weight). On day 5, auranofin-treated chicken embryos were 100% protected against death (*p* = 0.0002) and had a significantly reduced parasite load as determined by histopathology, immunohistochemistry and by the number of parasites quantified by real-time PCR.

**Conclusions:**

These results reveal *in vitro* and *in vivo* activity of auranofin against *T. gondii*, suggesting that it may be an effective alternative treatment for toxoplasmosis.

## Author Summary

*Toxoplasma gondii* is a protozoan parasite that infects at least two thirds of the world human population. Once it infects the human host, it has great predilection for the brain and the retina of the eye. It remains latent until the host's immune system weakens, and then causes organ tissue damage. There are very few treatments available that are active against this parasite, and they all fail to eradicate it from the human body. Hence, there is always a risk for recurrence and/or disabling long-term complications such as blindness or neurological abnormalities. Despite this fact, it has been over fifty years since most anti-*Toxoplasma* agents were initially described. Most recently, in an attempt to expedite the process of drug discovery, older drugs are making a comeback by being re-purposed for new diseases. Auranofin, which was originally designed to treat rheumatoid arthritis, has consistently shown antiparasitic activity against multiple organisms, including parasites of great public health importance such as *Plasmodium*, *Schistosoma* and *Leishmania*, although most of these reports are based on *in vitro* assays. Herein, we present our studies that demonstrate that auranofin is active against *Toxoplasma gondii in vitro* and in an animal model of acute *Toxoplasma* infection, suggesting that auranofin has great potential to become a new anti-*Toxoplasma* agent.

## Introduction

*Toxoplasma gondii* is the second leading cause of hospitalizations (8%) and deaths (24%) among foodborne pathogens in the US. People typically become infected by three principal routes of transmission: foodborne, animal-to-human (zoonotic) and mother-to-child (congenital), and rarely as post-solid organ transplant, blood transfusion or work related injuries. The number of primarily infected individuals varies widely worldwide: 22.5% of the American population is infected with this parasite [1], while in other parts of the world, the infection prevalence can be as high as 95%. These individuals are at risk of developing disease which usually follows after congenital transmission or reactivation of *T. gondii* latent forms (bradyzoites) in immunocompromised hosts [2]. Unfortunately, current available therapies have significant toxicity and are only active against one lifecycle stage of the parasite, the tachyzoite, and have no effect over the bradyzoite form [3], [4]. Furthermore, the impending threat of emergence of resistance to these therapies makes the discovery of new therapeutic targets a priority.

One promising re-profiled drug, auranofin, a gold containing compound that is FDA approved for the treatment of rheumatoid arthritis, has recently shown broad antiparasitic activity against *Plasmodium falciparum* [5], *Leishmania infantum* [6], *Schistosoma mansoni* [7] and *Entamoeba histolytica* [8] among others. Auranofin's anti-parasitic activity seems to stem from its gold molecule that readily dissociates and targets thioredoxin reductase, which we have recently demonstrated in our work with *Entamoeba histolytica* trophozoites [8] and cysts of *Entamoeba invadens* (manuscript in preparation). Given that thioredoxin reductase is a highly conserved enzyme in protozoan parasites [9] and based on our preliminary data, we hypothesized that auranofin has activity against *T. gondii*.

## Materials and Methods

### Ethics statement

Per Public Health Services (PHS) Policy, the Institutional Animal Care and Use Committee (IACUC) oversight is not required for egg model of toxoplasmosis using unhatched eggs. PHS Policy is applicable to proposed activities that involve live vertebrate animals. While embryonal stages of avian species develop vertebrae at a stage in their development prior to hatching, Office for Protection from Research Risks (OPRR) has interpreted "live vertebrate animal" to apply to avians (e.g., chick embryos) only after hatching (http://www.gpo.gov/fdsys/pkg/CFR-2009-title9-vol1/xml/CFR-2009-title9-vol1-chapI-subchapA.xml).

### Host cells and parasite cultures

Primary human foreskin fibroblasts (HFF) were initially cultured in Dulbecco's modified Eagle's medium supplemented with 10% fetal bovine serum (Gibco, Life Technologies, Carlsbad, Calif.), penicillin and streptomycin (50 µg/ml each) and maintained subsequently in the same medium with 2% fetal bovine serum (FBS). *T. gondii* RH tachyzoites (National Institutes of Health AIDS Reference and Reagent Repository, Bethesda, MD) and RH tachyzoites expressing cytoplasmic yellow fluorescent protein (YFP, kindly provided by M.J.Gubbels, Boston College, Boston, Massachusetts) [10] were maintained by serial passage in HFF monolayers at 37°C in a humid 5% CO_2_ atmosphere.

### Drug treatment

Auranofin (Enzo Life Sciences), was dissolved in 100% ethanol as a stock solution (4 mg/mL) and then diluted in complete tissue culture medium (DMEM +2% FBS) for final concentrations of 0.1 to 19 µM. For *in vivo* experiments, the auranofin concentration used was 1 mg/kg of estimated body weight.

Pyrimethamine (Sigma Aldrich) was dissolved in 100% ethanol in a stock concentration of 5 mg/mL. Three final dilutions in complete tissue culture medium were examined: 0.02, 0.1 and 0.2 µM. Sulfadiazine (Sigma Aldrich) was dissolved in complete medium at a final stock concentration of 5 mg/mL. Three doses in complete medium were evaluated: 0.2, 1 and 2 µM. Testing of pyrimethamine/sulfadiazine combinations by checkerboard method (using abovementioned concentrations) was carried out in triplicates and in three independent experiments.

### Growth monitoring by fluorescence

Black, 96-well, tissue culture-treated plates with optical clear bottoms were purchased from Greiner bioOne (Germany). HFF cells were added to wells in a final volume of 200 µl and grown to confluence. Freshly syringed, lysed parasites were filtered through a 5- µm polycarbonate filter (sterile Millex SV low binding durapore PVDF syringe filters), centrifuged, and resuspended in parasite culture medium without phenol red (Gibco, Life Technologies, Carlsbad, Calif.). HFF host cells were infected with 0.5×10^3^ YFP- expressing RH tachyzoites in the presence of different dilutions of auranofin, sulfadiazine, pyrimethamine, or control (ethanol alone) in triplicate. Plates were kept in a humidified incubator with 5% CO_2_ at 37°C for five days. After 5 days of incubation, plates were washed, fixed with 4% paraformaldehyde and read with a Synergy Mx BioTek (Vermont, US) multimode microplate reader Gen5 Software (excitation 510 nm; emission 540 nm). For excitation, a single flash from a UV Xenon lamp was used for each well, and emission signals were recorded with a sensitivity setting of 100. The values are presented as percentages of growth inhibition relative to the untreated controls (defined as 100% survival).

### Modified plaque assays

YFP-expressing RH tachyzoites, grown in DMEM 2% FBS without phenol red, syringed, lysed and filtered as described above, were used to infect 6-well plates containing fresh, confluent HFF monolayers. Each well was inoculated with 0.5×10^3^ YFP-expressing RH tachyzoites. The treatment group was treated with auranofin (0.4 µM), and the parasites were allowed to grow for seven days before fixation with 4% paraformaldehyde. Plaques were defined as independent foci of green fluorescence that correspond to a cluster of YFP tachyzoites infecting multiple adjacent HFFs. These plaques were visualized and counted per low power field (20x) with an inverted fluorescence microscope.

### Cell viability assays

CellTiter 96 Non-Radioactive Cell Proliferation Assay was performed according to the manufacturer's instructions (Promega). Confluent monolayers of HFF host cells (approximately 1×10^4^) plated in clear bottom 96-well plates were treated with multiple dilutions of auranofin (0.1–19.2 µM). After 5 days incubation, 15 µL of dye solution (tetrazolium salt) was added to each well and the plates were incubated at 37°C for 4 hr. The Solubilization Solution/Stop Mix was then added to the culture wells to solubilize the formazan product, and the absorbance at 570 nm was recorded using a 96-well plate reader (Synergy Mx BioTek (Vermont, US) multimode microplate reader Gen5 Software). The 570 nm absorbance reading is directly proportional to the number of cells normally used in proliferation assays. The values are presented as percentages relative to the untreated controls (defined as 100% survival).

### Invasion and replication assays

In preparation for invasion and replication assays, RH wild type tachyzoites were grown for 72 hrs. Intracellular parasites were collected as described above. Monolayers of confluent HFF cells were grown in 8-well chamber slides. Three independent experiments were conducted with triplicates, and at least 100 cells were counted. The number of infected cells and the number of tachyzoites per vacuole was determined per each high power field by light microscopy.

#### Invasion assays

HFF cell monolayers were infected with 0.5×10^6^ parasites per well. The treatment group was infected in the presence of auranofin at a final concentration of 0.4 µM. After 1 hour of infection, monolayers were washed, fixed with methanol, and stained with Giemsa stain.

#### Replication assays

Parasites were grown and collected as for the invasion assays. Monolayers of confluent HFF cells grown in 8-well chamber slides were infected with 0.05×10^6^ RH wild type tachyzoites and incubated for 4 hours. After incubation, monolayers were washed and complete medium (DMEM +2% FBS) was added back to the wells. Treatment groups received auranofin at a final concentration of 0.4 µM. After 24 hrs incubation, monolayers were washed and fixed with methanol for subsequent Giemsa staining.

### Viability of extracellular parasites by ATP assays

The effect of auranofin on tachyzoites of RH wild type *T. gondii* was assessed by ATP assays. Extracellular parasites (0.25×10^6^ per experimental group) collected as previously described, were kept in suspension with complete medium (DMEM +2%FBS) with or without auranofin (0.4 µM) for 0, 2, 4, 6, 8 hrs and 18 hrs (overnight). After incubation, experimental groups were sonicated, aliquots were spun down at 4000 rpm for 5 min, and supernatants extracted for ATP assays. CellTiter-Glo Luminescent Cell Viability Assay was performed according to manufacturer's instructions (Promega). Fifty microliters of supernatant from each experimental group were aliquoted into wells in a 96 opaque-wells plate (Nunc) in triplicate. Subsequently, an equal volume of CellTiter-Glo Reagent was added to each well. Stabilization of the luminescence was accomplished by incubating the plate for 10 mins at room temperature. Readings were performed with a GloMax Luminometer (Promega). The readings are presented as relative luminescence units (RLU).

### Chicken embryo model of acute toxoplasmosis

We have previously standardized the chicken embryo model [11]. Briefly, twelve day old pathogen-free fertilized chicken eggs (McIntyre Farms, Hemet, CA) were incubated at 37°C in a humid incubator. At 14-days old, a small window was cut through the shell with a hand drill directly over the blood vessel in each egg, and the vein was visualized with 1 drop of sterile mineral oil on the exposed membrane. Tachyzoites (1×10^4^) in Dulbecco's modified Eagle's medium with or without auranofin (1 mg/kg of predicted weight for age) were injected directly into the chorioallantoic vein [11] with a 28-gauge needle without pre-incubation. The windows were sealed with tape, and the embryos were incubated in a 37°C incubator. The eggs were candled once a day daily to assess viability. Livers and brains were harvested from the embryos either at the time of their death or 5 (or 8) days post-infection, whichever occurred first. One half of each collected organ was fixed in 4% paraformaldehyde for histopathology (hematoxylin and eosin staining and for immunohistochemistry staining for *T. gondii* with anti-*T. gondii* HRP antibody). The second half of each organ was frozen at −70°C for subsequent quantitative PCR analysis.

### Quantification of *T. gondii* by real-time PCR

To quantify tachyzoites *in vivo*, a standard curve was constructed by adding 10^7^ tachyzoites to brain or liver samples (100 mg) from 19-day-old chicken embryos and homogenizing the preparation with a cordless homogenizer (VWR) in cell lysis solution. Total genomic DNA was extracted from 25 mg tissue aliquots with the Qiagen DNeasy Blood & Tissue Kit per manufacturer's instructions (Qiagen, Alameda, CA). DNA was eluted in 200 µl of DNA elution buffer, and then serially diluted to create a standard curve [11]. Tissue from experimental embryos was similarly harvested and genomic DNA extracted as above. Using 2 µl aliquots of eluted genomic DNA as template, quantitative PCR amplification was performed to determine the relative amount of *T. gondii* surface antigen (SAG1) gene, a constitutively produced gene. Quantitative PCR was performed in duplicate using primers 5′-GTC ATT GTA GTG GGT CCT TCC-3′ and 5′-GCC TCA TCG GTC GTC AAT AA-3′ and PrimeTime probe 5′-TCC TAC GGT GCA AAC AGC ACT CTT-3′ (IDT), and the cycling conditions were 95°C for 10 min, followed by 40 cycles of 95°C for 15 s, and 60°C for 1 min. The relative amount of product generated was measured by determining the threshold cycle when the level of specific PCR product as measured by probe fluorescence that exponentially increased and crossed the threshold of a passive reference dye (ROX) in each sample. The standard curves (with known numbers of tachyzoites added to uninfected liver or brain) were used to extrapolate the numbers of tachyzoites present in unknown samples. Results are presented as relative log_10_ of relative numbers of tachyzoites per organ.

### Statistical analysis

Results were analyzed using GraphPad Prism software 6.0. All the *in vitro* experiments and the qPCR quantification results were analyzed with two-tailed, non paired, non-parametric tests to determine statistically significant differences (*p*<0.05; CI 95%) between control and treatment groups. A Kaplan Meier survival curve was calculated comparing control *vs.* chicken embryos treated with a single dose of auranofin.

Response data measurements were fit to a sigmoid Emax model using the computer program NONMEM ver 7.2 (ICON, Dublin, Ireland). A naïve-pooled approach was employed incorporating all individual experiment results in the analysis.

## Results

### Potency determination of anti-toxoplasmic effect of auranofin

**Figure 1:**
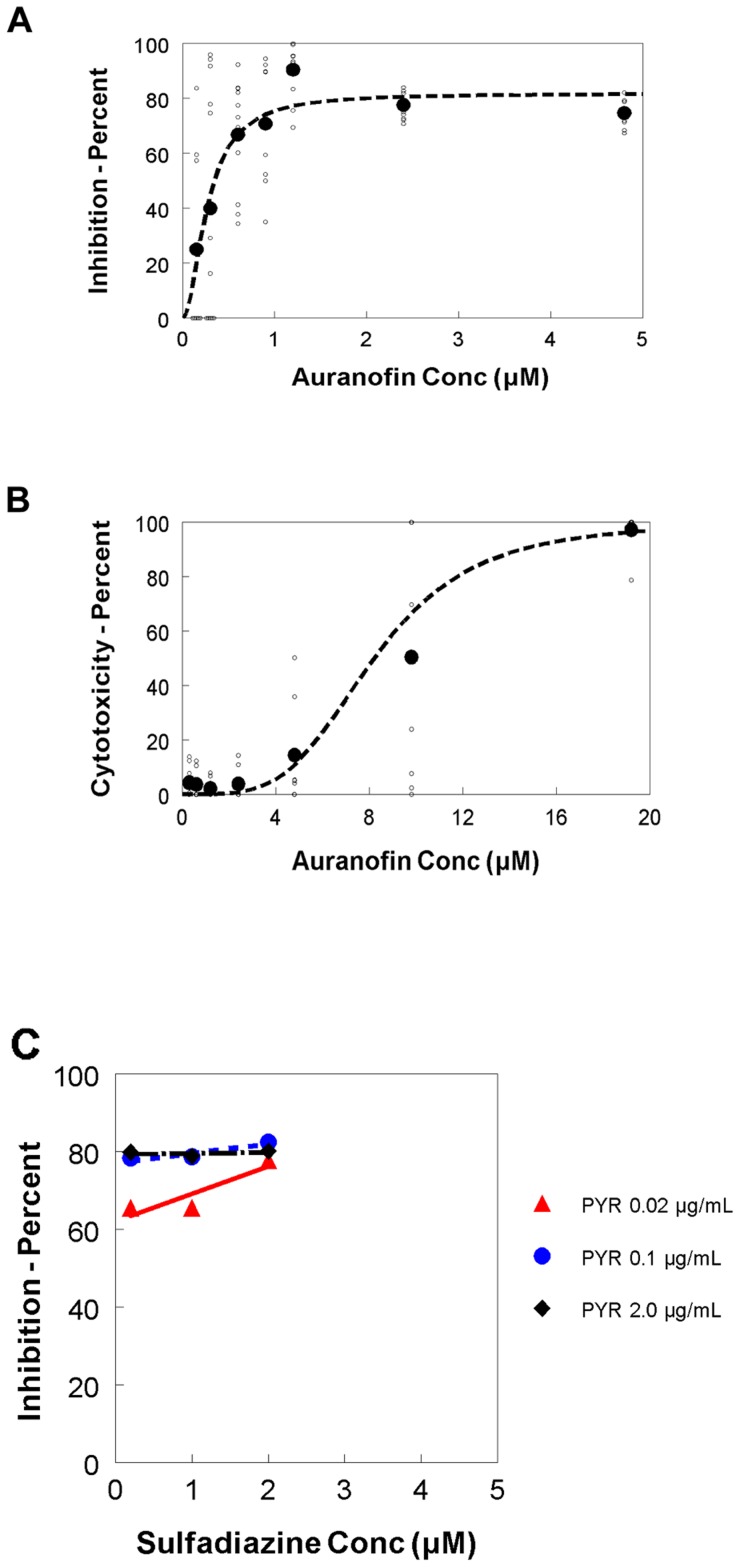
Auranofin effect on the growth of YFP-expressing RH Toxoplasma gondii tachyzoites. **A.** The percentage of growth inhibition of all observations from 3 independent experiments was plotted for serial two-fold dilutions of auranofin. The different concentrations of auranofin are presented in the X axis. (IC_50_ = 0.28 µM). **B.** Cytotoxicity assays showed that auranofin does not affect HFF viability at anti-parasitic doses (TD_50_  = 8.21 µM for HFFs). **C**: The percentage of growth inhibition of all observations from 3 independent experiments was plotted for combinations of pyrimethamine/sulfadizine by checkerboard method (pyrimethamine doses: 0.02, 0.1 and 0.2 µM; sulfadiazine doses: 0.2, 1 and 2 µM).

For IC_50_ determination experiments, 96-well plates (clear bottoms) with confluent monolayers of HFF cells were infected with 0.5×10^3^ YFP-RH tachyzoites for 4 hrs. At the end of infection period, extracellular parasites were removed and complete medium (DMEM +2% FBS without phenol) was added back with twofold serial dilutions of auranofin yielding a concentration range of 0.15–4.8 µM. Fluorescence measurements at day 5 post infection showed that auranofin inhibited growth by 50% at a concentration of 0.28 µM (IC_50_) (**Figure 1A**, Hill coefficient = 1.94; maximum response or Emax: 82%). In comparison, all different combinations of pyrimethamine and sulfadiazine generated a maximum response of 80% (Emax: 80%; Figure 1C).

### Screen for cytotoxic effect of auranofin on HFF host cells

For host cell toxicity assays, 96-well plates with confluent monolayers of HFFs host cells were treated with twofold serial dilutions of auranofin yielding a concentration range of 0.3–19.2 µM. Triplicates per experimental group were read at 120 hrs (Day 5). By measuring absorbance at day 5 post-infection, auranofin caused cell cytotoxicity in 50% of the cells at a concentration of 8.21 µM (TD_50_) (Figure 1B, Hill coefficient 3.89).

### *In vitro* effects of auranofin on *T. gondii*

#### Effects on invasion and replication

**Figure 2:**
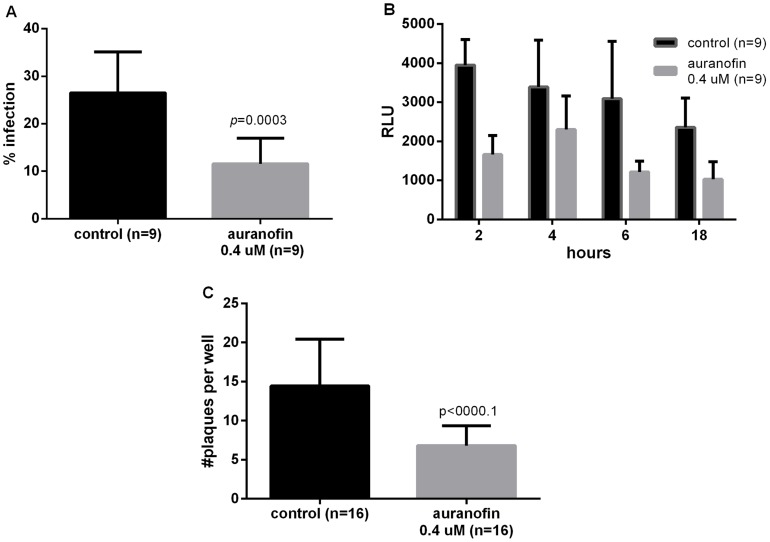
Effect of auranofin on parasite viability and plaque formation. **A.** Auranofin significantly decreased the rate of infection after 24(three independent experiments were conducted with triplicates, and at least 100 cells were counted. The number of infected cells was determined per each high power field by light microscopy. The percentage of infection was calculated as the total number of infected cells divided by the total number of cells multiplied by 100). **B.** Viability of extracellular tachyzoites in complete medium at 18 hrs (overnight) using ATP assay. RLU: relative luminescence units. **C.** The number of plaques observed at day 7 post infection was significantly different. Observations from 3 independent experiments are presented (n = total number of observations). Data presented as mean ±SD.

At 0.4 µM, auranofin inhibited replication (as measured by % infected fibroblasts at 24 hrs): 10.94% *vs.* 24.66% of controls (*p* = 0.0003) (**Figure 2A**) with no effect on parasite invasion (16.95% *vs.* 12.91% *p* = 0.4331). No differences in the number of parasites per infected cell either in the invasion or replication assays were observed (data not shown).

#### Direct effects of auranofin on *T. gondii* viability

Viability of extracellular parasites treated with auranofin at 0, 2, 4, 6, and 18 hrs (overnight) was assessed by ATP assays. For those parasites treated with auranofin at 0.4 µM, *T. gondii* viability was affected after 2 hrs of exposure to auranofin, and it became maximal after overnight incubation (18 hrs): 62% of parasites from the treatment group were non-viable compared to the control group as per ATP viability assay (*p* = 0.0003) (**Figure 2B**).

#### Plaque assays

HFFs infected with YFP tachyzoites and treated with auranofin (0.4 µM), exhibited significantly fewer plaques compared to control after 7 days of incubation post infection (12.5 *vs.* 7.5; *p* = 0.0003; n = 16 per group) (**Figure 2C**).

### *In vivo* effect of auranofin in a chicken embryo model of acute toxoplasmosis

**Figure 3:**
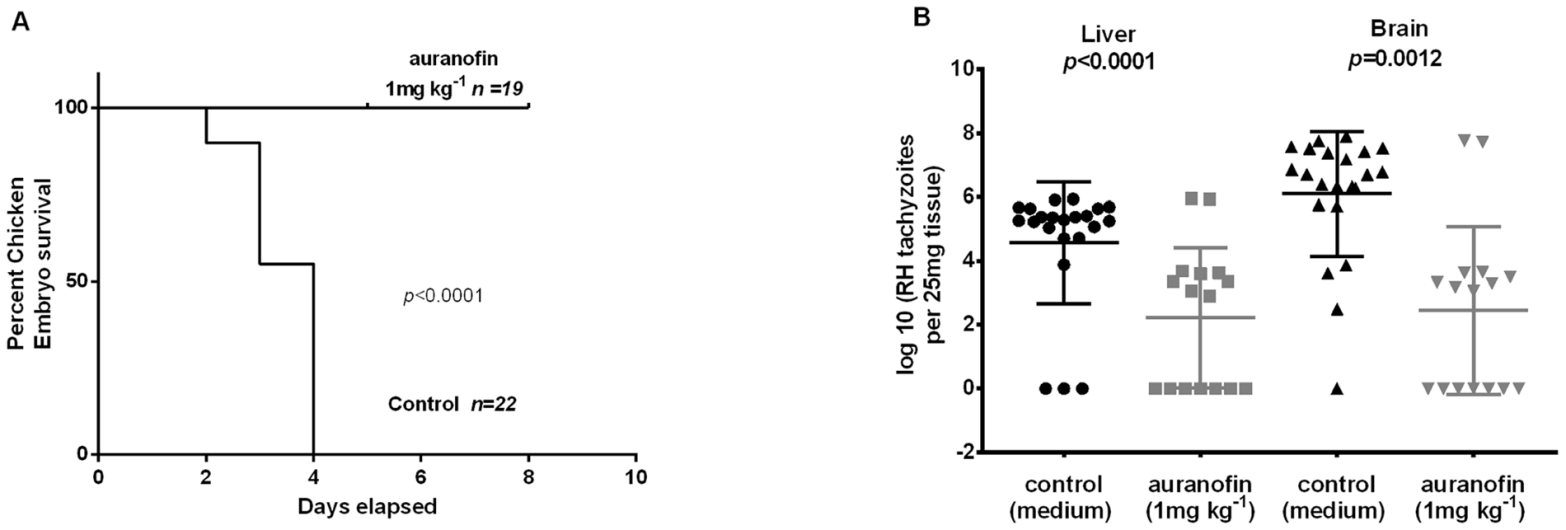
Effect of auranofin on the survival of chicken embryos infected with RH T. gondii tachyzoites- model of acute toxoplasmosis. **A.** Kaplan Meier survival curve of infected chick embryos over 5 days after treatment with auranofin with a single dose of 1 mg/kg. Additional subjects that were allowed to incubate for 8 days are included in this survival curve. **B** Quantification of parasite load (mean ±SD) in brains and livers of chicken embryos presented as the log_10_ of number of tachyzoites as determined by real-time PCR per 25 gram of tissue. Control chicken embryos: 22; auranofin-treated embryos: 19*. * Three chicken embryos that died on the day of injection due to bleeding were not included in the experiment.

**Figure 4:**
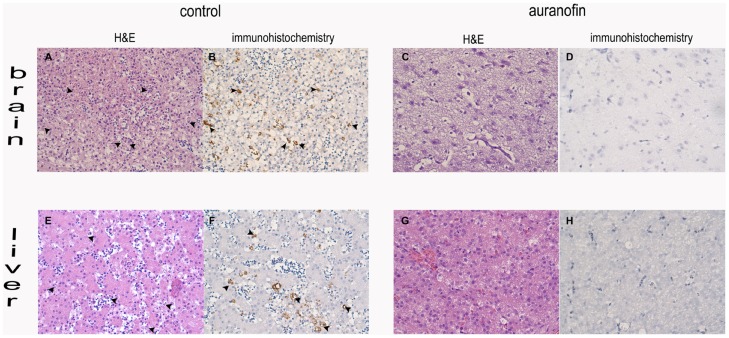
Hematoxylin & eosin histopathology and immunohistochemistry of brains and livers. Arrows point at parasites. A, B, E, F: control tissue with substantial amount of inflammation. C, D,G,H: tissue from auranofin-treated embryos.

Fourteen day old chicken embryos were injected through the chorioallantoic vein with 1×10^4^ tachyzoites of the virulent RH strain. The treatment group received auranofin at the time of inoculation at a dose of 1 mg/kg (estimated body weight). While all control embryos died by day 4, auranofin-treated chicken embryos were 100% protected against death by day 5 (*p* = 0.0002) (**Figure 3A**) and had a significantly reduced parasite load as determined by histopathology and by the number of parasites quantified by real-time PCR (expressed as a log_10_) from their brains (5.27 *vs* 2.98; *p* = 0.002) and livers (6.705 *vs* 3.11; *p* = 0.0003) (**Figure 3B**) and histopathology and immunohistochemistry (**Figure 4**). Of note, the amount of tissue decomposition found in the control chicken embryos suggested that they died one day before their documented death (on day 3).

## Discussion

We demonstrate for the first time, that auranofin has significant activity against *T. gondii*. *In vitro*, auranofin reduced parasite replication, while *in vivo*, it reduced the parasite load and most remarkably, only one dose of auranofin prevented death in a model of acute toxoplasmosis. Our study of the anti- *T. gondii* effects of auranofin *in vitro* showed that it affects parasite viability, reducing its replication ability without affecting its capacity to invade the host cell in the absence of host cell toxicity. These results are compelling since auranofin is a FDA approved drug with a known safety profile, which can expedite its use in clinical trials.

Auranofin is active against *T. gondii* similarly to the activity described for other protozoans of great public health importance such as *Plasmodium falciparum* [5] and *Leishmania infantum* [6]. In our study, our Emax modeling of the treatment response to auranofin demonstrated a Hill coefficient greater than 1, suggesting positive co-operativity or independent binding of auranofin to its target. Additionally, auranofin's maximum inhibition of *T. gondii* growth was 82%, which is equivalent to the effect observed with current standard of therapy for toxoplasmosis (all sulfadiazine and pyrimethamine combinations, across the board, generated a calculated maximum effect of 80%) (Figure 1C).

Auranofin had a TD_50_ for HFF host cells that was 29 fold higher than the IC_50_ of 0.28 µM suggesting a high therapeutic index. This therapeutic index supports its safety as a potential alternative treatment for acute *T. gondii* infection.

The independent IC_50_ of sulfadiazine (26.05 µM) and pyrimethamine IC_50_ (0.402 µM), as demonstrated by Meneceur et al [12] are higher than that of auranofin (0.28 µM). However, the combination of lower doses of pyrimethamine and sulfadiazine demonstrated a maximum inhibitory effect over the growth of *T. gondii* of 80% consistently throughout multiple combinations (Figure 1C). Auranofin's maximum inhibitory effect was equivalent to that of sulfadiazine-pyrimethamine (Figure 1A and 1C). This suggests that auranofin could be an alternative candidate for the treatment of acute toxoplasmosis, although further studies are needed before consideration of clinical trials.

Auranofin's anti-toxoplasmic mechanism of action is not known. However, from our *in vitro* studies we can surmise that auranofin affects replication while it does not exert any effect during the *T. gondii* dynamic invasion process. We challenged HFFs with *T. gondii* tachyzoites for 5, 15 and 30 min and found no differences in invasion (data not shown). Even when the invasion time was prolonged to 1 hr, we did not detect any difference in the rate of invasion whether cells were treated or not with auranofin. Contrary to these findings, we observed statistically significant differences in the percentage of infected cells after overnight incubation post-infection in the presence of auranofin as compared to controls. This latter observation strongly suggests that auranofin affects replication of the parasite by inhibiting its growth.

On the other hand, the molecular target of auranofin, as an antiparasitic agent, is strongly suggested by the current literature. Angelucci *et al* [7], demonstrated that auranofin inhibited *Schistosoma mansoni* glutathione-thioredoxin reductase, which the parasite solely relies on for antioxidant protection. Similarly, we recently reported that *Entamoeba histolytica* was rendered vulnerable to auranofin's antiparasitic effect because it inhibits its sole thioredoxin reductase [8]. As an intracellular parasite, *T. gondii* needs to circumvent host cell-mediated oxidant attacks during its invasion and replication; therefore, it is conceivable that *T. gondii* thioredoxin reductase might be the target for auranofin. However, *T. gondii* possesses multiple anti-oxidant enzymes that might be directly or indirectly affected by auranofin: thioredoxin reductase, glutathione reductase and thioredoxin-dependent peroxidases [13]. Other targets within the dynamic parasitophorous vacuole (which results from the direct interaction between the parasite and the host cell) are also unknown. We performed assays to differentiate the load of reactive oxygen species (ROS) with dichlorodihydrofluorescein between auranofin-treated and control cells. Although we observed differences between control and auranofin-treated infected cells per fluorescence microscopy, we failed to demonstrate quantifiable differences (data not shown). Further studies are underway to determine the exact molecular target and mechanism of action involved in auranofin's anti-*Toxoplasma* activity.

The most striking results came from our *in vivo* chicken embryo model of acute toxoplasmosis. All chicken embryos treated with a single dose of auranofin survived to the end of the experiment (after 5 days post infection), while all the control subjects succumbed to overwhelming infection no later than day 3 post infection (**Figure 3A**). Similar effects were observed if the embryos were injected on day 12 (instead of day 14) and were allowed to incubate for 8 days post infection (data included in the survival curve). Hence, a single dose of auranofin provided 100% protection from death in all acutely infected embryos, while it reduced the parasite load in organs such as the brain and the liver with almost no inflammatory reaction associated with the lower parasite load (see hematoxylin & eosin histology in **Figure 4**). Although, parasites were not completely absent in the auranofin-treated group, the observed 100% *in vivo* survival was achieved with only one dose of auranofin. We cannot demonstrate the viability of the parasites detected in the treatment group since we used DNA detection by qPCR. Further studies with mouse animal models with a standard daily treatment regimen are part of our immediate future studies.

One of the limitations of this chicken embryo model is its short course. The chicken embryo is not allowed to hatch, hence we are not able to prolong incubation beyond 21 days (5–8 days post infection).Chicken embryos inoculations with *T. gondii* tachyzoites at stages earlier than 12 days old are technically challenging given the fragility of their blood vessels, which is also the reason why repeated doses of auranofin are not possible.

In contrast, the standard mouse animal model of acute toxoplasmosis requires at least 10 days of daily therapy and subsequent post-treatment follow up in order to determine the efficacy of the study drugs to eradicate parasite load and ensure survival of the mice. We are planning further experiments in this standard model in the future.

Given its effect on both the parasite and the host, auranofin stands out as a unique anti-parasitic agent: it can protect sanctuary organs such as the brain, where the host‘s own protective inflammatory responses might cause further organ damage. Additional pharmacokinetic studies for auranofin in the CNS are necessary in order to establish its bioavailability in the setting of an abnormally permeable blood brain barrier during a CNS infection. This is particularly important, since most pharmacokinetic studies on auranofin were performed in the early ‘80 s [14]–[16] in uninfected animals with normal blood brain barriers.

In summary, these results reveal significant *in vitro* and *in vivo* activity of auranofin against *T. gondii*, suggesting that it may be an effective alternative treatment for acute toxoplasmosis in the future.
